# Matters Arising: PREDICT underestimates survival of patients with HER2-positive early-stage breast cancer

**DOI:** 10.1038/s41523-023-00514-5

**Published:** 2023-03-16

**Authors:** Ahmed M. Alaa, Adrian L. Harris, Mihaela van der Schaar

**Affiliations:** 1 University of California, Berkeley, Berkeley, CA USA; 2 University of California, San Francisco, San Francisco, CA USA; 3 University of Oxford, Oxford, UK; 4 University of Cambridge, Cambridge, UK; 5 University of California, Los Angeles, Los Angeles, CA USA

**Keywords:** Breast cancer, Risk factors

**arising from** Elisa Agostinetto et al. *NPJ Breast Cancer* 10.1038/s41523-022-00452-8 (2022)

In a recent paper in *NPJ Breast Cancer*, Agostinetto et al.^1^ demonstrated the poor concordance between recently improved survival data for HER2-positive early-stage breast cancer with outcomes predicted by PREDICT 2.1. We replicated these findings in large-scale cohorts extracted from the UK and US patient registries and demonstrated that a publicly available machine learning-based prognostic model provides improved predictive accuracy.

We do need improved prognostic tools that issue reliable predictions across all patient subgroups, and so we developed a publicly available predictive model, Adjutorium (https://adjutorium-breastcancer.herokuapp.com/), using an advanced automated machine learning algorithm to predict individual breast cancer mortality, all-cause mortality and benefit from adjuvant therapies^2^. The model makes predictions based on seven patient-specific variables: age at diagnosis, ER status, HER2 status, tumour grade, number of lymph nodes, tumour size, and whether the tumour was detected via routine screening or symptoms. We trained and internally validated the model using data for 395,862 patients from the UK National Cancer Registration and Analysis Service (NCRAS). Because of differences in drug access and practice, we then externally validated the model using data for 571,635 patients from the US Surveillance, Epidemiology, and End Results (SEER) Program.

**Fig. 1:**
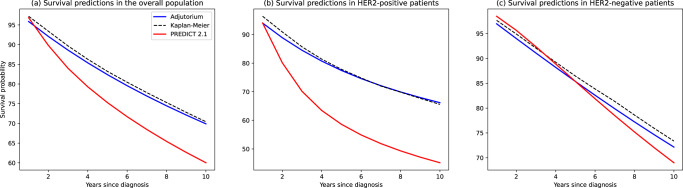
Comparison between the average survival predictions issued by Adjutorium and PREDICT. **a**–**c** The predicted survival curves by the two models for the overall population, the HER2-positive and HER2-negative subtypes, respectively. The average survival curve predicted by Adjutorium is colored in blue, whereas the survival curve issued by PREDICT is colored in red. The empirical Kaplan–Meier survival curves are depicted through the dashed black lines. These evaluations were conducted using the NCRAS registry data by computing the predicted survival curve issued by the two models for each patient and averaging over the population.

Our validation experiments show that PREDICT exhibits patterns of survival underestimation that bear some resemblance to those reported by Agostinetto et al. In particular, PREDICT underestimated 5-year survival in patients with HER2-positive breast cancer by 16% (Fig. 1). On the contrary, Adjutorium underestimated survival in the same cohort by only 0.9%. Unlike the analysis conducted by Agostinetto et al. using the ALTTO data, we found that PREDICT underestimates survival in HER2-positive patients more severely than in HER2-negative patients, in which PREDICT underestimated survival by 1%. These discrepancies might be a result of the differences in the time period over which data was collected in our analysis cohort compared to the ALTTO trial (both the internal and external data sets comprised breast cancer diagnoses spanning the years 2000–2016), as well as the differences between the treatment regimens used in our observational cohort and those used in the trial data. However, the observation that PREDICT underestimates survival in HER2-positive patients remains consistent across both studies.

**Fig. 2:**
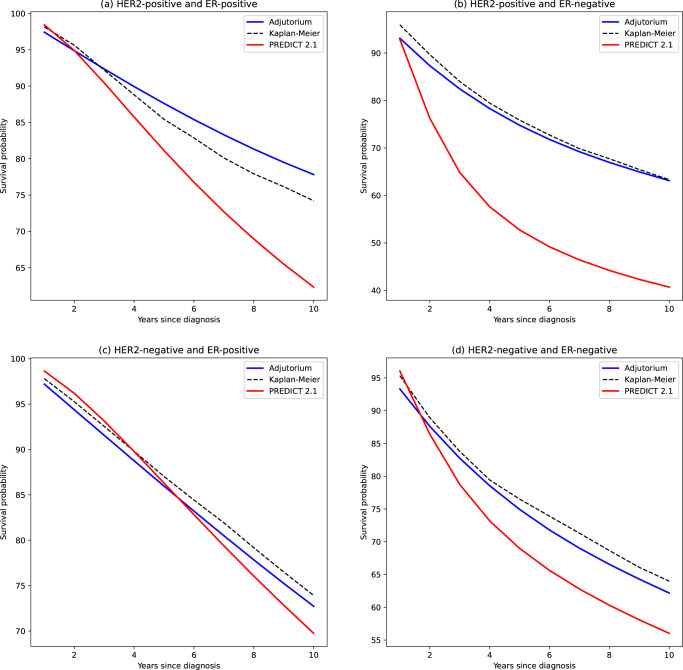
Comparison between the average survival predictions issued by Adjutorium and PREDICT in different breast cancer subtypes. **a**–**d** The predicted survival curves by the two models for patients with HER2-positive/ER-positive, HER2-positive/ER-negative, HER2-negative/ER-positive and HER2-negative/ER-negative, respectively. The survival curve predicted by Adjutorium is colored in blue, whereas the survival curve issued by PREDICT is colored in red. The empirical Kaplan–Meier survival curves are depicted through the dashed black lines. These evaluations were conducted using the NCRAS registry data.

The prognostic accuracy improvements achieved by Adjutorium were not just in the HER2-positive patients but also across all subtypes of patients stratified by HER2 and ER status (Fig. 2). Most notably, in patients with ER-positive breast cancer, survival estimates of PREDICT were underestimated by 23% as compared to 7% for Adjutorium. In the overall population of patients involved in our large-scale study, PREDICT underestimated survival by 5.47% as opposed to 0.94% for Adjutorium. In both the internal and external validation cohorts, Adjutorium and PREDICT v2.1 disagreed on the treatment decisions. In all comparisons, Adjutorium showed significantly greater ROC curves and better concordance with the population data. Treatment decisions informed by Adjutorium are less likely to over- or under-treat patients^2^. The improved accuracy of Adjutorium is likely a result of using flexible (nonparametric) machine learning models, more recent patient data and a larger sample size compared to PREDICT.

In order to improve accessibility of our model, we provided an easy-to-use online tool for breast cancer prediction based on the Adjutorium model (https://adjutorium-breastcancer.herokuapp.com/), where patient features can be easily input to a graphic interface that survival time with different treatment options. The interface style will be familiar to users of PREDICT v2.1, but ours is an open-source software that enables other researchers to easily re-fit the model as more data becomes available.

Validation of prognostic models using data from clinical trials is crucial for evaluating the clinical utility of these models in informing therapeutic decisions. Both PREDICT v2.1 and our model were derived from retrospective data with confounding factors that influence both patient outcomes and treatment assignments. Some of these confounding factors might be unobserved (e.g., patient-level socio-economic variables), which might undermine the quality of prognostic predictions issued by these models. This might be a possible explanation for why PREDICT exhibits a significant underestimation of survival outcomes. Clinical trials resolve any biases induced by confounding by randomising treatment assignments. Hence, it would be very useful if Dr. Agostinetto and her colleagues would consider validating Adjutorium in the ALTTO trial data to test whether its prognostic gains still hold in patient data that does not suffer from confounding.
